# Design and development of a disease-specific quality of life tool for patients with aplastic anaemia and/or paroxysmal nocturnal haemoglobinuria (QLQ-AA/PNH)—a report on phase III

**DOI:** 10.1007/s00277-019-03681-3

**Published:** 2019-05-21

**Authors:** Cathrin Niedeggen, Susanne Singer, Martha Groth, Andrea Petermann-Meyer, Alexander Röth, Hubert Schrezenmeier, Britta Höchsmann, Tim H. Brümmendorf, Jens Panse

**Affiliations:** 1 Department of Oncology, Hematology, Hemostaseology and Stem Cell Transplantation, Medical Faculty, University Hospital RWTH Aachen, Pauwelsstr. 30, 52074 Aachen, Germany; 2 Division of Epidemiology and Health Services Research, Institute of Medical Biostatistics, Epidemiology and Informatics, University Medical Centre Mainz, Mainz, Germany; 3 Department of Hematology, University Hospital Essen, University of Duisburg-Essen, Essen, Germany; 4 Institute of Clinical Transfusion Medicine and Immunogenetics, German Red Cross Blood Transfusion Service and University Hospital Ulm and Institute of Transfusion Medicine, University of Ulm, Ulm, Germany

**Keywords:** Aplastic anaemia, Paroxysmal nocturnal haemoglobinuria, Quality of life, Bone marrow failure syndromes

## Abstract

To date, instruments to measure quality of life (QoL) specifically for patients with acquired aplastic anaemia (AA) and paroxysmal nocturnal haemoglobinuria (PNH) are lacking altogether. As a consequence, this issue is either underevaluated or alternatively, instruments originally designed for cancer patients are being used. We therefore started to systematically develop a AA/PNH-specific QoL (QLQ-AA/PNH) instrument in these ultra-rare diseases according to European Organisation for Research and Treatment of Cancer (EORTC) guidelines. While phases I and II of the process have previously been published, we now report on the resulting instrument (phase III of this process). As part of the phase III of the evaluation process, we approached patients through physicians, patient support groups, and patient conferences. After participants completed the preliminary questionnaire and reported socio-demographic data, they were interviewed in person or via phone with a debriefing interview to find out whether the items were relevant, easy to understand, and acceptable to patients and whether there was anything missing in the questionnaire. We hypothesised what items could be combined into a scale and calculated Cronbach’s alpha to define its preliminary internal consistency. After definition of a priori criteria to keep or delete items, a group of six experts met in person, discussed the results, and decided on in- or exclusion. A total of 48 patients were enrolled, 21 of those suffered from AA (44%), 13 from PNH (27%), and 14 from AA/PNH syndrome (29%). The median time to complete the 69 items was 10 min (range 5–20), mean time 11 min. The compliance criterion (> 95% completion) was fulfilled by 57 items. Twenty-three items were mentioned as especially relevant by ≥ 2% of the patients. Cronbach’s alpha of the hypothesised scales ranged from 0.63 (social support) to 0.92 (fear of progression and illness intrusiveness). Finally, 47 items were kept; 16 were deleted, and 5 were changed, while 1 item expanded. This resulted in 54 items in total. As no issues were mentioned to lacking by a minimum of five patients, no items were added to the questionnaire. After completion, the AA/PNH-QoL tool (QLQ-AA/PNH) was translated according to EORTC guidelines into English, French, and Italian. For patients with PNH and AA until now, the standard assessment for QoL was to use the EORTC Quality of Life Questionnaire (QLQ-C30) or the Functional Assessment of Chronic Illness Therapy Fatigue Instrument (FACIT-Fatigue). We herewith present a new instrument aimed to be better tailored to the needs of PNH and AA patients. The anticipated fourth development phase will be performed for psychometric validation; however, we already explored the internal consistency of the hypothesised scales and found the results to be very good. Hence, the new QLQ-AA/PNH with 54 items can be used in trials and clinical studies from now on, according to EORTC strategy even if the scoring algorithm at this point is preliminary and the QLQ-AA/PNH might change slightly after phase IV. This is important, as there are no other disease-specific instruments available for AA/PNH patients right now.

## Introduction

Quality of life (QoL) assessment is an essential patient-reported outcome to evaluate the effects and value of treatment [1]. For the evaluation of QoL in cancer patients, the European Organisation for Research and Treatment of Cancer Quality of Life Core Questionnaire (EORTC QLQ-C30) [2] is a widely accepted tool. It has additional modules for several malignancies such as head and neck cancer, multiple myeloma, breast cancer, and others in order to better target and reflect disease-specific problems [2–8]. Modification of the EORTC QLQ-C30 also became necessary with the advent of new treatment modalities such as antibodies and tyrosine kinase inhibitors, as these highly effective treatments lead to new side effects and hitherto unrecognised psychosocial QoL effects e.g. in patients with chronic myeloid leukaemia [9]. Hence, as QoL instruments become more and more disease- and treatment-specific, this trend, so far, pertains only malignant and well-known diseases.

However, about 8000 known rare and ultra-rare diseases affect a substantial proportion of patients worldwide [10]. For these patients, it can often be difficult to find an experienced specialist or institution, let alone an approved treatment. It is even more difficult to find a reliable QoL tool, correctly assessing QoL or treatment effects and value [11] thus allowing to properly adjust treatment and care to the needs of these patient and foster patient-centred care.

Within the broad area of non-malignant haematology, both acquired aplastic anaemia (AA) and paroxysmal nocturnal haemoglobinuria (PNH) represent interrelated ultra-rare diseases with a yearly incidence within the western hemisphere of 1.3 to 2 per million [12, 13]. The exact number of patients newly developing AA, PNH, or AA/PNH overlap syndromes e.g. within Germany is unknown. The incidence in Germany is estimated to be 250 per year, but given the fact that not all of the affected patients are diagnosed properly and timely, the actual number of newly diagnosed patients is assumed to be somewhat lower. AA and PNH often affect young patients. While age distribution for AA shows a bimodal curve with a peak in young adults, the mean age at diagnosis of PNH is somewhat higher and peaks between 30 and 45 years. PNH and AA belong to the group of bone marrow failure syndromes (BMFS) and must be regarded as two distinct but interrelated manifestations of a partly common pathophysiology [14, 15].

For patients with AA, treatment options include allogeneic bone marrow transplantation (BMT) from sibling donors, immunosuppressive therapy (IST) including anti-thymocyte-globulin (ATG) preferentially from horse, further courses of IST, BMT from alternative donor sources, thrombopoietin (TPO) receptor agonists [16], and experimental treatment approaches [13, 17, 18].

Treatment with the complement inhibitor eculizumab is regarded as the treatment of choice for symptomatic and/or predominantly haemolytic PNH patients, which greatly increased survival rates for these patients [19–23].

In contrast to the vast amount of published research addressing pathophysiology and treatment of AA and PNH, QoL, and psychosocial issues have not been intensively studied. Almost all QoL reports in PNH used the EORTC QLQ-C30 [2] or the Functional Assessment of Chronic Illness Therapy Fatigue Instrument (FACIT-Fatigue) [24, 25], and both questionnaires are now routinely used within the international PNH-registry [26]. QoL reports about AA patients mostly focus on sequelae from bone marrow transplantation [27–29] and rarely concentrate on non-transplanted patients [30, 31].

In addition, evaluations mainly used surrogate parameters e.g. treatment toxicity, transfusion and drug treatment requirements, and haematologic counts in order to indirectly describe quality of life in patients with AA/PNH.

The lack of specific QoL tools in patients with AA and/or PNH and the strong encouragement by patient advocacy groups led to the development of a AA/PNH-specific QoL (QLQ-AA/PNH) instrument according to EORTC-guidelines [32]. The study’s objective was not to measure QoL and compare it between different groups of patients but to develop a questionnaire that is able to serve such a purpose in patients with AA and/or PNH. For this, it is necessary to include patients with various disease stages and treatment regimens, different levels of education, different age, gender etc. In fact, the sample should be as heterogeneous as possible, according to international guidelines [32].

Phases I and II of the developmental process have been published already [33]; herein, we report on phase III. Apart from validation in routine clinical practice, a particular aim of the phase III in this ultra-rare disease condition was to identify and solve potential problems in the administration of the questionnaire e.g. the phrasing of questions, and to identify missing or redundant items. We also wanted to obtain mean response scores for each item to determine their relevance.

## Patients and methods

The data protection official of the University Hospital RWTH Aachen and the Institutional Review Board of the Medical Faculty RWTH Aachen University approved the study protocol.

### Sampling and data collection

We approached patients via specialised physicians known to work within German reference centres for patients with AA/PNH, patient support groups, their AA/PNH website, and during two patient conferences (in Ulm and Essen, Germany). In addition, patients themselves spread the word through social media such as Facebook and encouraged each other to participate in the study so that patients approached the authors via email or telephone. Patients were eligible if they had been diagnosed with AA, PNH or both, and if they had not participated in previous phases I–II of this module development. Each participant provided written informed consent.

Patients were asked to report about issues related to their disease not their treatment-specific problems e.g. after allogeneic stem cell transplantation or drug-associated problems (e.g. virilisation due to cyclosporine). Further information can be found here [33].

After the first 18 patients, an unexpected rush of patients approached us after presentation of the project at one of the patient conferences. We therefore decided to change the process as the subsequent 30 patients lived all across Germany.

That is, we send out the questionnaire to the patients. Patients were then interviewed through telephone twice. They first were given instructions and information about the aim of the study and the questionnaire and were allowed to ask questions. They then filled out the questionnaire taking the time they needed to do so. They were then called again in order to give their feedback and to discuss remaining issues. The same author (C.N.) conducted all the telephone interviews. We did not formally evaluate differences between answers given from patients interviewed via telephone vs. patients interviewed in person; however, we did not perceive any differences in answers, time of interviews or mood during the interviews.

### The instruments

The preliminary questionnaire which was developed in phases I and II included 77 questions. Before starting with phase III, these were reduced to 69 questions by merging highly related issues such as activities of daily living and employment. This was done through consensus by the research team.

With the resulting 69 questions, the participants were asked first to complete the preliminary questionnaire and to report socio-demographic and clinical characteristics, such as education, employment, disease, and treatment history.

After that, they were interviewed in person by one of the authors (C.N.) with debriefing questions regarding the time needed to complete the questionnaire. They could also indicate whether they found any item upsetting, confusing, or particularly (ir)relevant. If they mentioned any problems, they were asked to suggest an alternative wording for that item. After the first nine patients, minimal modifications in wording were incorporated; questions were arranged in a slightly different order, and another nine patients went through the same procedure.

All Information regarding disease and treatment were reported by the patients.

### Statistical analyses

First, the items s25, s27, s45, s46, s49, s66, s67, and s68 were recoded so that higher values indicate more problems, to ensure comparability with the other items.

We then calculated the range and mean of each item together with the percentage of responses in each response category. In a next step, we defined for each item the percentage of patients who completed it, who found it irritating, or difficult to understand.

We hypothesised what items could be combined into a scale and calculated Cronbach’s alpha to define its preliminary internal consistency.

All analyses were performed using STATA statistical software, version 12 (StataCorp, TX, USA).

### Decision on items

We defined a priori criteria when to keep an item in the questionnaire. An item should be kept if at least five of the following criteria 1 to 7 or criterion 8 is fulfilled:

1. Relevance: mean > 1.5 (of a Likert response scales 1 to 4)
2. Relevance: > 50% of the participants score 3 (“quite a bit”) or 4 (“very much”)
3. No floor nor ceiling effects: > 10% of the patients score 1 or 2; > 10% score 3 or 4
4. Range: the responses range from 1 to 4
5. Acceptability: < 5% find the item upsetting
6. Easiness: < 5% find the item difficult to understand
7. Compliance: > 95% complete the item
8. Priority: ≥ 2% mention the item as especially relevant

The results per item were tabulated accordingly. A group of six experts met in person, discussed the results, and decided on in- or exclusion of items. They based their decision on these criteria but they could also deviate from it if they had strong arguments, for example, based on clinical experience, and if they found a consensus. The group was composed of physicians (C.N., B.H., A.R, T.H.B., and J.P.) and a psychologist (S.S.).

If at least five patients mentioned the same issue as lacking from the questionnaire, it was to be added.

## Results

### Sample characteristics

A total of 48 patients were enrolled, 17 via their physicians, 20 from patient conferences, 10 via the AA-PNH website, and 1 via another patient (word of mouth). The disease was AA in 21 cases (44%), PNH in 13 (27%), and mixed AA+PNH in 14 (29%). The participants were 23 to 83 years old at the time of the study; 49 years on average (mean) (see Table 1 for more details). The time from first symptom(s) to first doctor visit ranged from few days to 4 years (median 0 weeks, mean 13 weeks). The time from first doctor visit to diagnosis ranged from 1 week to 7 years (median 6 weeks, mean 25 weeks). In five cases, the diagnosis was made accidentally after a routine check-up or other pre-operative routine differential blood counts taken. At the time of diagnosis, the patients were 14 to 77 years old (mean 40 years). As this was a “mixed bag” of patients with some having received IST, some stem cell transplantation, some complement inhibition, and some a sequence of more than one, steroids had been given to most of the patients at some point in time.

**Table 1:** Sample/patient characteristics (*n* = 48)

		*N*	%
Disease	AA	21	44%
	PNH	13	27%
	AA+PNH	14	29%
Treatment	Cyclosporin A	34	71%
	Eculizumab	20	42%
	Transfusions	33	68%
	Steroids	28	57%
	Stem cell transplantation	8	17%
ATG	No ATG	25	52%
	Horse	11	23%
	Rabbit	6	13%
	Horse or rabbit	3	6%
	ATG not further specified	3	6%
Sex	Male	19	40%
	Female	29	60%
Age at the time of interview	In years (median; mean; range)	47; 49; 23–83	
Age at diagnosis	In years (median; mean; range)	37; 40; 14–77	
Time from first symptoms until first physician contact (in months)	In months (median; mean; range)	0; 13; 0–208	
Time from first physician contact to final diagnosis	In months (median; mean; range)	6; 25; 1–364	

*ATG* anti-thymocyte globulin

### Completion of the questionnaire

The time to complete the 69 items ranged from 5 to 20 min (median 10 min, mean 11 min). The compliance criterion (> 95% completion) was fulfilled by 57 items (see Table 2 for details).

**Table 2:** Item characteristics and decision of expert group

			Crit 1		Crit 2		Crit 3		Crit 4		Crit 5		Crit 6		Crit 7		Crit 8	Crit 1–7 or crit 8	
Item number	Wording	Mean	Mean > 1.5?	Scores 3/4	Scores 3/4 > 50%?	Scores 1/2	Neither floor nor ceiling	Range	Range	% upsetting	< 5% upsetting	% difficult	< 5% difficult	Compliance	> 95% complete?	% preferred	≥ 2% preferred		Group decision
*s01*	Have you felt dizzy?	1.8	1	0.23	0	0.77	1	1 to 4	1	0.00	1	0.00	1	1.00	1	0.00	0	1	*Delete, because symptom is rare*
*s02*	Have you been physically restless?	2.0	1	0.30	0	0.70	1	1 to 4	1	0.00	1	0.05	0	1.00	1	0.00	0	1	*Delete, because symptom is rare and some patients did not understand it*
*s03*	Have you been exhausted?	2.9	1	0.66	1	0.34	1	1 to 4	1	0.00	1	0.00	1	1.00	1	0.00	0	1	*Delete, too similar to s06*
s04	Did you need to rest?	2.7	1	0.57	1	0.43	1	1 to 4	1	0.00	1	0.00	1	1.00	1	0.00	0	1	Keep
s05	Were you exhausted for several days after exertion?	2.1	1	0.34	0	0.66	1	1 to 4	1	0.00	1	0.00	1	1.00	1	0.00	0	1	Keep
s06	Were you tired?	2.9	1	0.64	1	0.36	1	1 to 4	1	0.00	1	0.00	1	1.00	1	0.00	0	1	Keep
s07	Did you have difficulties getting out of bed in the morning?	2.4	1	0.38	0	0.62	1	1 to 4	1	0.00	1	0.00	1	1.00	1	0.00	0	1	Keep
s08	Did your body feel heavy?	2.1	1	0.34	0	0.62	1	1 to 4	1	0.00	1	0.07	0	0.96	1	0.00	0	1	Keep
s09	Were you short of breath?	2.1	1	0.36	0	0.64	1	1 to 4	1	0.00	1	0.00	1	1.00	1	0.00	0	1	Keep
s10	Did it bother you that you had to pay attention to small symptoms, in case they could indicate something serious?	2.1	1	0.30	0	0.70	1	1 to 4	1	0.00	1	0.00	1	1.00	1	0.00	0	1	Keep
s11	Have you experienced problems with an increased tendency to bleed?	1.7	1	0.21	0	0.79	1	1 to 4	1	0.00	1	0.00	1	1.00	1	0.00	0	1	Keep
s12	Have you been more susceptible to infection/s?	1.9	1	0.23	0	0.77	1	1 to 4	1	0.00	1	0.00	1	1.00	1	0.00	0	1	Keep
s13	Have you experienced swelling or inflammation in your mouth?	1.8	1	0.26	0	0.74	1	1 to 4	1	0.00	1	0.00	1	1.00	1	0.00	0	1	*Change: have you experienced problems with swelling or inflammation in your mouth?*
s14	Did you find it difficult to take a long stroll/walk?	2.1	1	0.34	0	0.66	1	1 to 4	1	0.00	1	0.00	1	1.00	1	0.00	0	1	Keep
s15	Have you had difficulties climbing stairs?	2.4	1	0.43	0	0.57	1	1 to 4	1	0.00	1	0.00	1	1.00	1	0.00	0	1	Keep
*s16*	Have you had problems doing sports?	2.5	1	0.51	1	0.43	1	1 to 4	1	0.00	1	0.00	1	0.94	0	0.00	0	1	*Delete, because applicable only to few patients, better in s26*
s17	Have you had difficulties standing for a longer period of time?	1.9	1	0.30	0	0.70	1	1 to 4	1	0.00	1	0.00	1	1.00	1	0.00	0	1	Keep
s18	Have you suffered from sleep disturbance?	2.3	1	0.36	0	0.64	1	1 to 4	1	0.00	1	0.00	1	1.00	1	0.00	0	1	Keep
s19	Have you felt impaired by pain in your daily life?	1.6	1	0.17	0	0.83	1	1 to 4	1	0.00	1	0.00	1	1.00	1	0.00	0	1	Keep
s20	Have you had problems managing your household tasks?	1.9	1	0.23	0	0.74	1	1 to 4	1	0.00	1	0.00	1	0.98	1	0.00	0	1	Keep
*s21*	Did you find it difficult to find a balance between over- and underchallenge?	2.2	1	0.36	0	0.60	1	1 to 4	1	0.00	1	0.07	0	0.96	1	0.02	1	1	*Delete, difficult to understand and the issue is covered in other items*
s22	Was it a burden to you that you had to ration your energy?	2.3	1	0.40	0	0.60	1	1 to 4	1	0.00	1	0.00	1	1.00	1	0.02	1	1	Keep
s23	Have you lacked the strength for your private life and hobbies?	2.1	1	0.32	0	0.64	1	1 to 4	1	0.00	1	0.02	1	0.96	1	0.00	0	1	Keep
s24	Were you limited at work or during any other daily activity?	2.3	1	0.40	0	0.57	1	1 to 4	1	0.00	1	0.00	1	0.98	1	0.00	0	1	Keep
s25	Have you been able to achieve what you wanted to?	3.1	1	0.79	1	0.19	1	1 to 4	1	0.00	1	0.00	1	0.98	1	0.02	1	1	Keep
s26	Was it a burden to you to have to abstain from sports?	1.9	1	0.21	0	0.68	1	1 to 4	1	0.00	1	0.00	1	0.89	0	0.00	0	1	Keep
s27	Were you able to go on vacation the way that you wanted to?	3.1	1	0.72	1	0.21	1	1 to 4	1	0.00	1	0.00	1	0.94	0	0.05	1	1	*Change of time frame (0.5 year)*
s28	Did it bother you not being able to make plans ahead of the time?	2.6	1	0.47	0	0.45	1	1 to 4	1	0.00	1	0.00	1	0.91	0	0.05	1	1	Keep
s29	Have you been unable to get up the energy to do anything, or have you felt sluggish?	2.2	1	0.36	0	0.62	1	1 to 4	1	0.00	1	0.00	1	0.98	1	0.00	0	1	Keep
s30	Did it bother you that you had to be cautious?	2.2	1	0.36	0	0.62	1	1 to 4	1	0.00	1	0.00	1	0.98	1	0.02	1	1	Keep
*s31*	Did it bother you that you had to be reasonable?	2.2	1	0.36	0	0.60	1	1 to 4	1	0.00	1	0.07	0	0.96	1	0.00	0	1	*Delete, too similar to s30*
s32	Was your normal rhythm of life disturbed?	2.3	1	0.38	0	0.60	1	1 to 4	1	0.00	1	0.00	1	0.98	1	0.02	1	1	Keep
s33	Did it bother you not being able to be spontaneous?	2.3	1	0.40	0	0.51	1	1 to 4	1	0.00	1	0.02	1	0.91	0	0.00	0	1	Keep
*s34*	Did it bother you that you could not act flexibly?	2.6	1	0.47	0	0.38	1	1 to 4	1	0.00	1	0.05	0	0.85	0	0.00	0	0	*Delete, difficult to understand and too similar to s33*
s35	Have you had difficulties concentrating?	2.3	1	0.36	0	0.62	1	1 to 4	1	0.00	1	0.00	1	0.98	1	0.00	0	1	Keep
s36	Did you feel irritable?	2.2	1	0.32	0	0.66	1	1 to 4	1	0.02	1	0.00	1	0.98	1	0.02	1	1	Keep
s37	Did you always have to take care not to catch any infections?	2.4	1	0.40	0	0.55	1	1 to 4	1	0.00	1	0.00	1	0.96	1	0.00	0	1	Keep
*s38*	Did you try to avoid things that could cause any infections?	2.3	1	0.38	0	0.57	1	1 to 4	1	0.00	1	0.00	1	0.96	1	0.05	1	1	*Delete, better in s37*
*s39*	Did you feel distressed because of limited life perspective?	2.5	1	0.47	0	0.51	1	1 to 4	1	0.02	1	0.00	1	0.98	1	0.02	1	1	*Delete, better in s40*
s40	Have you felt burdened by thoughts about an uncertain future?	2.6	1	0.47	0	0.47	1	1 to 4	1	0.02	1	0.02	1	0.94	0	0.02	1	1	Keep
*s41*	Have you been “out of order” due to the illness?	2.4	1	0.45	0	0.53	1	1 to 4	1	0.00	1	0.00	1	0.98	1	0.02	1	1	*Delete, not sensitive to change and better covered by other items*
s42	Has everything revolved around your illness?	2.2	1	0.34	0	0.62	1	1 to 4	1	0.00	1	0.00	1	0.96	1	0.02	1	1	Keep
s43	Did you have the feeling you were missing out on life?	2.4	1	0.51	1	0.47	1	1 to 4	1	0.00	1	0.00	1	0.98	1	0.02	1	1	Keep
*s44*	Have you felt you were not moving forward?	2.3	1	0.45	0	0.53	1	1 to 4	1	0.00	1	0.02	1	0.98	1	0.02	1	1	*Delete, because not clear enough*
*s45*	Has empathy of friends and family helped you?	2.6	1	0.60	1	0.40	1	1 to 4	1	0.00	1	0.00	1	1.00	1	0.00	0	1	*Delete, too similar to s46*
s46	Has support of friends and family helped you?	2.3	1	0.49	0	0.51	1	1 to 4	1	0.00	1	0.00	1	1.00	1	0.00	0	1	*Change: did you feel supported by friends and family?*
s47	Have you suffered, because your environment has been burdened by your illness?	2.7	1	0.51	1	0.47	1	1 to 4	1	0.00	1	0.00	1	0.98	1	0.00	0	1	Keep
s48	Did it bother you to constantly be confronted with your illness?	2.9	1	0.64	1	0.34	1	1 to 4	1	0.00	1	0.00	1	0.98	1	0.00	0	1	Keep
s49	Were you proud of what you achieved despite the illness?	2.4	1	0.49	0	0.51	1	1 to 4	1	0.00	1	0.00	1	1.00	1	0.00	0	1	Keep
*s50*	Did it bother you not being treated in daily life as everybody else?	1.9	1	0.13	0	0.70	1	1 to 4	1	0.00	1	0.07	0	0.83	0	0.00	0	0	*Delete, because relevance differs per patient*
s51	Did it burden you to be labelled “sick“?	2.3	1	0.38	0	0.62	1	1 to 4	1	0.00	1	0.00	1	1.00	1	0.02	1	1	Keep
s52	Has it frustrated you that you have had to justify yourself as to why, for example, you were unable to do something?	2.3	1	0.38	0	0.51	1	1 to 4	1	0.00	1	0.00	1	0.89	0	0.02	1	1	Keep
s53	Have you feared a deterioration in your blood results?	2.8	1	0.62	1	0.38	1	1 to 4	1	0.00	1	0.00	1	1.00	1	0.02	1	1	Keep
s54	Have you felt burdened by your blood results?	2.5	1	0.51	1	0.49	1	1 to 4	1	0.00	1	0.00	1	1.00	1	0.02	1	1	Keep
s55	Have you been afraid that treatments might fail?	2.5	1	0.51	1	0.49	1	1 to 4	1	0.00	1	0.00	1	1.00	1	0.02	1	1	Keep
s56	Have you been afraid there would be no treatments any more for you?	2.4	1	0.43	0	0.55	1	1 to 4	1	0.00	1	0.00	1	0.98	1	0.02	1	1	*Change: have you been concerned that there would be no more viable treatment for you?*
*s57*	Have you feared a stem cell transplantation?	2.5	1	0.43	0	0.45	1	1 to 4	1	0.02	1	0.00	1	0.87	0	0.02	1	1	*Delete, too many missing; applicable only to few patients*
s58	Have you been afraid of relapse or deterioration?	2.8	1	0.60	1	0.38	1	1 to 4	1	0.00	1	0.00	1	0.98	1	0.05	1	1	Keep
s59	Did you feel vulnerable?	2.4	1	0.45	0	0.55	1	1 to 4	1	0.00	1	0.00	1	1.00	1	0.00	0	1	Keep
s60	Have visible signs of your illness (e.g. paleness, bruises, dark urine, yellow skin colour) constantly reminded you of your illness?	2.2	1	0.40	0	0.57	1	1 to 4	1	0.00	1	0.00	1	0.98	1	0.00	0	1	Keep
s61	Have you felt at the mercy of your illness?	2.4	1	0.40	0	0.55	1	1 to 4	1	0.00	1	0.00	1	0.96	1	0.00	0	1	Keep
s62	Did it bother you having to deal with the illness lifelong?	2.6	1	0.47	0	0.51	1	1 to 4	1	0.02	1	0.00	1	0.98	1	0.00	0	1	*Delete, too similar to other items*
s63	Have you worried a lot?	2.8	1	0.62	1	0.38	1	1 to 4	1	0.00	1	0.00	1	1.00	1	0.00	0	1	Keep
s64	Have you felt depressed?	2.4	1	0.45	0	0.55	1	1 to 4	1	0.00	1	0.00	1	1.00	1	0.00	0	1	Keep
s65	Did you feel less attractive because of your illness?	2.0	1	0.32	0	0.66	1	1 to 4	1	0.00	1	0.02	1	0.98	1	0.00	0	1	Keep
s66	Did you feel comfortable in your own body?	2.9	1	0.70	1	0.28	1	1 to 4	1	0.00	1	0.00	1	0.98	1	0.00	0	1	Keep
*s67*	Did you feel lighthearted?	3.4	1	0.83	1	0.13	1	1 to 4	1	0.00	1	0.02	1	0.96	1	0.00	0	1	*Delete, better in s63*
s68	Did it help you when you talked to other patients?	2.5	1	0.38	0	0.30	1	1 to 4	1	0.00	1	0.00	1	0.68	0	0.00	0	1	*Change: did you miss interaction with other patients? (time frame: 0.5 year)*
s69	For men: did you have problems with virility?	2.2	1	0.15	0	0.28	1	1 to 4	1	0.05	1	0.02	1	0.43	0	0.00	0	1	*Change: have you had less interest in sexuality? Could you enjoy sexuality less?*
Total			69		17		69		69		69		63		57		23	67	

Items that were changed or deleted are italicized. Items s25, s27, s45, s46, s49, s66, s67, and s68 were recoded. As described within the “Patients and methods,” a priori criteria when to keep an item in the questionnaire were defined (called “crit 1–8” within the table. An item should be kept if at least five of the following criteria 1 to 7 or criterion 8 is fulfilled:

1. Relevance: mean > 1.5 (of a Likert response scale 1 to 4)

2. Relevance: > 50% of the participants score 3 (“quite a bit”) or 4 (“very much”)

3. No floor nor ceiling effects: > 10% of the patients score 1 or 2; > 10% score 3 or 4

4. Range: the responses range from 1 to 4

5. Acceptability: < 5% find the item upsetting

6. Easiness: < 5% find the item difficult to understand

7. Compliance: > 95% complete the item

8. Priority: ≥ 2% mention the item as especially relevant

### Relevance and importance

All items had a mean score of > 1.5 and covered the full range, from 1 to 4. In 17 items, more than 50% of the participants indicated that they experienced this problem “quite a bit” or “very much.” None of the items had floor or ceiling effects. Twenty-three items were mentioned as especially relevant by ≥ 2% of the patients (Table 2).

### Acceptability and easiness

None of the items was rated as upsetting by more than 5% of the patients. However, the following six items were rated as being difficult to understand by at least 5% of the patients:

- Have you been physically restless?
- Did your body feel heavy?
- Did you find it difficult to find a balance between over- and underchallenge?
- Did it bother you that you had to be reasonable?
- Did it bother you that you could not act flexibly?
- Did it bother you not being treated in daily life as everybody else?

### Internal consistency

Cronbach’s alpha of the hypothesised scales ranged from 0.63 (social support) to 0.92 (fear of progression and illness intrusiveness). Other scales were fatigue (alpha = 0.88), infections (0.79), other symptoms (0.75), physical functioning (0.89), role functioning (0.82), emotional functioning (0.81), stigmatisation (0.78), and body image (0.82).

### Decision on items

Of all items, 67 fulfilled either criteria 1 to 7 or criterion 8. However, as a questionnaire with 67 items is relatively long; the group of experts went through all the items and identified similar items, where one of them could be deleted without reducing the comprehensiveness of the questionnaire. They also discussed changes of wording if needed. As a result, 47 items were kept; 16 were deleted, and 5 were changed, while 1 item expanded. This resulted in 54 items in total. The reasons for each change or deletion are documented in Table 2.

### Missing issues

No issues were mentioned as lacking by at least five patients. Hence, no items were added to the questionnaire.

## Discussion

The absence of a disease-specific QoL tool for patients with the ultra-rare diseases AA and/or PNH led to the development of an AA/PNH-specific QoL (QLQ-AA/PNH) instrument according to EORTC-guidelines [32]. Phases I and II of the process proved that despite the rareness of the diseases and the accompanying sparseness of patients, development of such a tool is feasible and both highly appreciated and actively supported by patients and patient advocacy groups [33]. It also showed that the EORTC-development guidelines cannot be followed in detail according to EORTC standard specification as e.g. large numbers of patients cannot be found within one hospital, not even within a treatment centre and gatherings of large groups of patients within one country are non-existent except for occasional rare patient conventions. We nonetheless succeeded to include 49 patients within the first two phases and were able to recruit another 48 patients for the phase III reported herein. Hence, a total of almost 100 patients with AA and/or PNH were recruited. As the main aim of this phase was to identify and solve potential problems in the administration of the questionnaire in order to come up with a concise, complete, and comprehensible QoL tool, patients had to be interviewed after working with the preliminary questionnaire. This turned out to be feasible though time-consuming for the first 18 patients but seemed unachievable later on when 30 more patients appeared within a short period of time from different areas all over Germany. Thus, we changed our study protocol after consultation with members of the EORTC Quality of Life Group (SS) and performed the last 30 interviews via telephone. Accrual of patients through patient advocacy groups and presentation of projects such as this at patient conferences, seems to be a potential blueprint for further studies of QoL within patient groups with ultra-rare diseases. Within other ultra-rare disease forms, QoL studies also used patient advocacy groups, international specialist, and the internet as a contact network in order to recruit relevant patient numbers [34, 35].

Our provisional questionnaire consisted of 69 items. The median time to complete this questionnaire was 10 min which seems to be a reasonable time for patients to fill out a QoL tool as it takes about 11 min to fill out the EORTC QLQ-C30 [36]. However, in order to achieve a questionnaire as fast as possible without compromising its comprehensiveness, this phase III study was performed and items difficult to understand or upsetting for the patients were removed. Moreover, we removed all items that seemed redundant. This resulted in a questionnaire with 54 items. As for patients with PNH until now, the standard assessment within clinical trials was to use the European Organisation for Research and Treatment of Cancer Quality of Life Questionnaire (EORTC QLQ-C30) [2] in addition to the Functional Assessment of Chronic Illness Therapy Fatigue Instrument (FACIT-Fatigue) [24, 25], which together sum up 46 items; thus, our QoL tool does not add a substantial number of items altogether. The psychometric properties of our new tool will be investigated in the final phase IV of this development process. However, we already explored the internal consistency of the hypothesised scales and found the results to be very good.

After completion of this phase III, the AA/PNH-QoL (QLQ-AA/PNH) tool was translated according to EORTC guidelines into English, French, and Italian [37]. The English version is depicted in Table 3. After completion of the contend validation within phase III, the fourth development phase will be performed for psychometric validation. However, validation usually relies on some form of intervention [2, 32, 36]. For patients with PNH and/or AA, interventional trials are rare. However, for PNH, currently, the number of clinical trials is increasing since new anti-complement strategies where developed and have just recently entered clinical trials [38, 39].

**Table 3:**
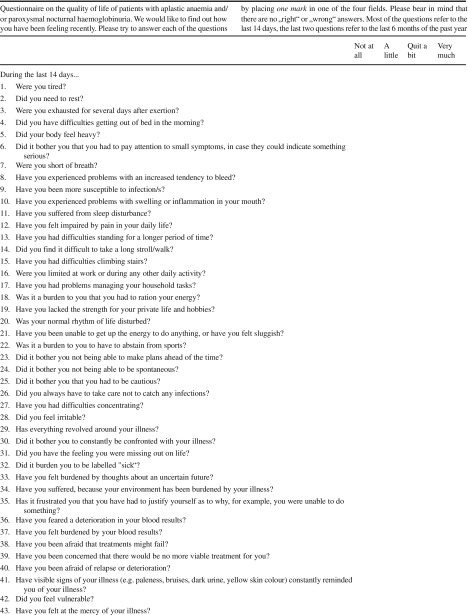

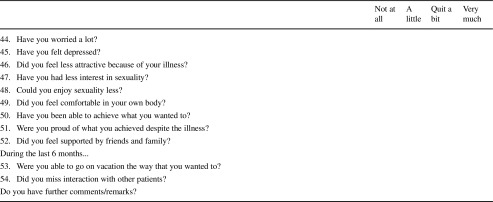
Provisional AA/PNH questionnaire.

We therefore chose a multi-pronged procedure in that we included the herein presented version of the QLQ-AA/PNH within the EMAA study (efficacy and safety of eltrombopag + CSA in patients with moderate aplastic anaemia; ClinicalTrials.gov identifier: NCT02773225) and added the AA/PNH-QoL tool to the smorgasbord of questionnaires, which are used within the PNH registry. This is an international registry established following a post-marketing commitment of the eculizumab manufacturer Alexion Pharmaceuticals Inc., requested by both the EMA (European Medicines Agency) and the FDA (Food and Drug Administration) prospectively documenting patients with PNH and PNH/AA overlap [26].

While the scoring algorithm at this point is preliminary and the QLQ-AA/PNH might change slightly after phase IV, it can be used in trials and clinical studies from now on, according to EORTC strategy. This is important, as there are no other disease-specific instruments available for AA/PNH patients right now. The only study, published just recently, is the one by Townsley et al. [40], in which eltrombopag in addition to standard IST was evaluated in AA patients. In there, patient-reported outcomes were measured with various tools such as the Patient-Reported Outcomes Measurement Information System (PROMIS) global physical health (GPH) and global mental health (GMH), functional assessment PROMIS sleep disturbance (Sleep) and applied cognitive abilities (Cog) in addition to other measures such as the cancer therapy–neutropenia 2 (FACT-N) [40–42]. This underscores the importance of our approach, as even in this study, various tools had to be used in order to better approach the patients’ quality of life.

As pointed out above, outcomes in studies with AA patients are almost universally not patient-reported and therefore should not be called QoL measurements while tools used for studies and the PNH registry are those which have been developed for cancer patients and have been shown not to cover QoL matters and issues of patients with a non-malignant ultra-rare haematologic disease [33].
